# Analyzing the regulation of miRNAs on protein-protein interaction network in Hodgkin lymphoma

**DOI:** 10.1186/s12859-019-3041-9

**Published:** 2019-09-02

**Authors:** Huimin Lei, Wenxu Liu, Jiarui Si, Ju Wang, Tao Zhang

**Affiliations:** 1 School of Biomedical Engineering, Tianjin Medical University, Tianjin, China; 2 School of Continuation Education, Tianjin Medical University, Tianjin, China; 3 School of Basic Medicine, Tianjin Medical University, Tianjin, China

**Keywords:** Protein interaction network, Network analysis, miRNA regulation, Hodgkin Lymphoma

## Abstract

**Background:**

Hodgkin Lymphoma (HL) is a type of aggressive malignancy in lymphoma that has high incidence in young adults and elderly patients. Identification of reliable diagnostic markers and efficient therapeutic targets are especially important for the diagnosis and treatment of HL. Although many HL-related molecules have been identified, our understanding on the molecular mechanisms underlying the disease is still far from complete due to its complex and heterogeneous characteristics. In such situation, exploring the molecular mechanisms underlying HL via systems biology approaches provides a promising option. In this study, we try to elucidate the molecular mechanisms related to the disease and identify potential pharmaceutical targets from a network-based perspective.

**Results:**

We constructed a series of network models. Based on the analysis of these networks, we attempted to identify the biomarkers and elucidate the molecular mechanisms underlying HL. Initially, we built three different but related protein networks, i.e., background network, HL-basic network and HL-specific network. By analyzing these three networks, we investigated the connection characteristic of the HL-related proteins. Subsequently, we explored the miRNA regulation on HL-specific network and analyzed three kinds of simple regulation patterns, i.e., co-regulation of protein pairs, as well as the direct and indirect regulation of triple proteins. Finally, we constructed a simplified protein network combined with the regulation of miRNAs on proteins to better understand the relation between HL-related proteins and miRNAs.

**Conclusions:**

We find that the HL-related proteins are more likely to connect with each other compared to other proteins. Moreover, the HL-specific network can be further divided into five sub-networks and 49 proteins as the backbone of HL-specific network make up and connect these 5 sub-networks. Thus, they may be closely associated with HL. In addition, we find that the co-regulation of protein pairs is the main regulatory pattern of miRNAs on the protein network in the HL-specific network. According to the regulation of miRNA on protein network, we have identified 5 core miRNAs as the potential biomarkers for diagnostic of HL. Finally, several protein pathways have been identified to closely associated with HL, which provides deep insights into underlying mechanism of HL.

**Electronic supplementary material:**

The online version of this article (10.1186/s12859-019-3041-9) contains supplementary material, which is available to authorized users.

## Background

Cancer is thought to be a kind of complex and highly heterogeneous disease that involves multiple causes and factors. Moreover, cancer is also associated with the alteration of molecular interactions rather than the abnormality of a single gene [1]. In particular, dysregulation of multiple pathways governing fundamental cell processes contributes to cancer development and progression. Therefore, these characteristics determine that we should apply systems biology approaches specifically network-based approaches to study underlying mechanism of cancer [2]. As protein-protein interactions (PPIs) form the basis of cellular processes, the dysfunction of some interactions causes many diseases including cancer [3]. Thus the construction and analysis of PPIs network can not only provide a global view of biological events, but also decipher the molecular basis of cancer from the perspective of network dynamics [4]. In addition, systematic analysis of the PPIs network also provide a wealth of valuable information that may be useful for identifying therapeutic targets [5, 6] and potential biomarkers for diagnosis and prognosis of cancer [7, 8].

As an important class of post-transcriptional regulator, microRNAs (miRNAs) can regulate many crucial cellular processes, such as differentiation, growth, proliferation, and apoptosis. The abnormality of miRNAs expression also leads to various diseases, especially cancer. It is well known that miRNAs play a crucial role in the formation and development of cancer by functioning as tumor suppressors or oncogene [9]. Moreover, miRNAs have also been considered as important molecules for cancer diagnosis [10] and therapeutic targets [11, 12].

miRNAs can negatively modulate target genes and consequently perform fine-scale adjustment of protein output by influencing the stability of encoding mRNAs [13]. In addition, miRNAs can also regulate functionally related proteins and exert specific effects on the formation of protein complexes [14, 15] and biological pathways [16]. Therefore, in order to more clearly understand the function of miRNAs and their role in diseases, the investigation of miRNA biology should be conducted in the context of protein interaction network rather than isolated target genes [17].

Although how miRNAs regulate protein interaction network is still not fully understood, some characteristics of miRNA-mediated protein interaction network have been investigated by integrating information about miRNA targets and protein interaction data [18, 19]. For instance, a statistical analysis was conducted to compare topological characteristics between miRNA-mediated proteins and randomly selected proteins from protein interactions network. The results demonstrated that the miRNA-mediated proteins tend to more frequently interact with other proteins. Moreover, the proteins mediated by the same miRNA have high tendency to interact with each other. These specific characteristics imply that miRNAs might exert their regulatory effects on protein complex and pathways through protein interactions network. Therefore, based on the analysis of miRNA-mediated protein interactions network, we can not more comprehensively understand the function of miRNAs [20], but more accurately identify the miRNAs associated with diseases [21, 22].

Hodgkin Lymphoma (HL) is a tumor arising from the lymphatic system and its hallmark is the emergence of Hodgkin and Reed-Sternberg cells [23]. Although the exact cause for HL is not clearly clarified yet, some risk factors have been considered to be related with the occurrence of HL. Because HL is an aggressive malignancy that can quickly spread through the body, identification of reliable diagnostic markers and efficient therapeutic targets are especially important for diagnosis and treatment of HL. Using the high-throughput techniques, many HL-related molecules have been identified, such as the proteins uniquely expressed in HL-derived cell lines [24] and miRNAs differently expressed between normal and patients with HL [25], which make it feasible to construct a specific network for HL. The analysis of such network can provide valuable insight into the underlying mechanism of HL and identification of key proteins and miRNAs for HL. For example, a regulatory network consisting of genes, miRNAs and transcription factors is constructed using the available data and several important pathways in HL are identified based on the resulting regulatory network [26]. However, this study just focused on the regulatory of miRNAs on isolated target genes and transcription factors. It is still unclear about the protein interactions network specific to HL and miRNA regulation on protein interactions network.

In this study, we firstly manually collected the HL-associated proteins and miRNAs. Subsequently, we extracted the experimentally verified protein-protein interactions from five protein interaction databases. Based on the collected data, we constructed a protein interactions network specific to HL using a three-step strategy. By analyzing this network, we identified the core proteins that are crucial for maintaining network structure. These proteins can be considered as candidates of diagnostic and therapeutic markers for HL. Finally, we obtained experimentally validated miRNA-target interactions from miRWalk and miRTarBase. By integrating HL-specific protein network with miRNA-target interactions, we investigate miRNA regulation on the HL-specific protein network. On the basis of the analysis at the network level, we obtain a comprehensive insight into the role of HL-associated proteins and miRNAs playing in pathogenesis of HL. These results provide more valuable information for studying mechanism and treatment of HL.

## Results

### Analysis of three related PPI networks

#### PPI background network

In order to provide a network-level view for the HL-specific proteins, we constructed a background network that includes as many proteins as possible. The constructed background network has 17,076 proteins as nodes and 146,295 protein interactions as edges. Subsequently, we calculated the degree distribution of the background network (shown in Additional file 1: Figure S1). As displayed in the figure, the degree distribution clearly follows a power law. It indicates that the background network is a typical scale-free network and has scale-free properties [27]. This result is also in agreement with the previous study [28].

The power-law decay of degree distribution implies that there are hub proteins that are heavily interacted with other proteins in the background network. In this study, we identified the hub proteins by calculating the relative connectivity of subgraph [29]. According to the previously study [30], the links between hub proteins in a network are systematically suppressed. Therefore, for the subgraphs consisting of only hub proteins, the relative connectivity will be smaller than that of other subgraphs containing non-hub proteins. Due to considering the unique topological property of hub proteins in the network, this identification method should be more precise compared with just using a degree threshold.

**Fig. 1:**
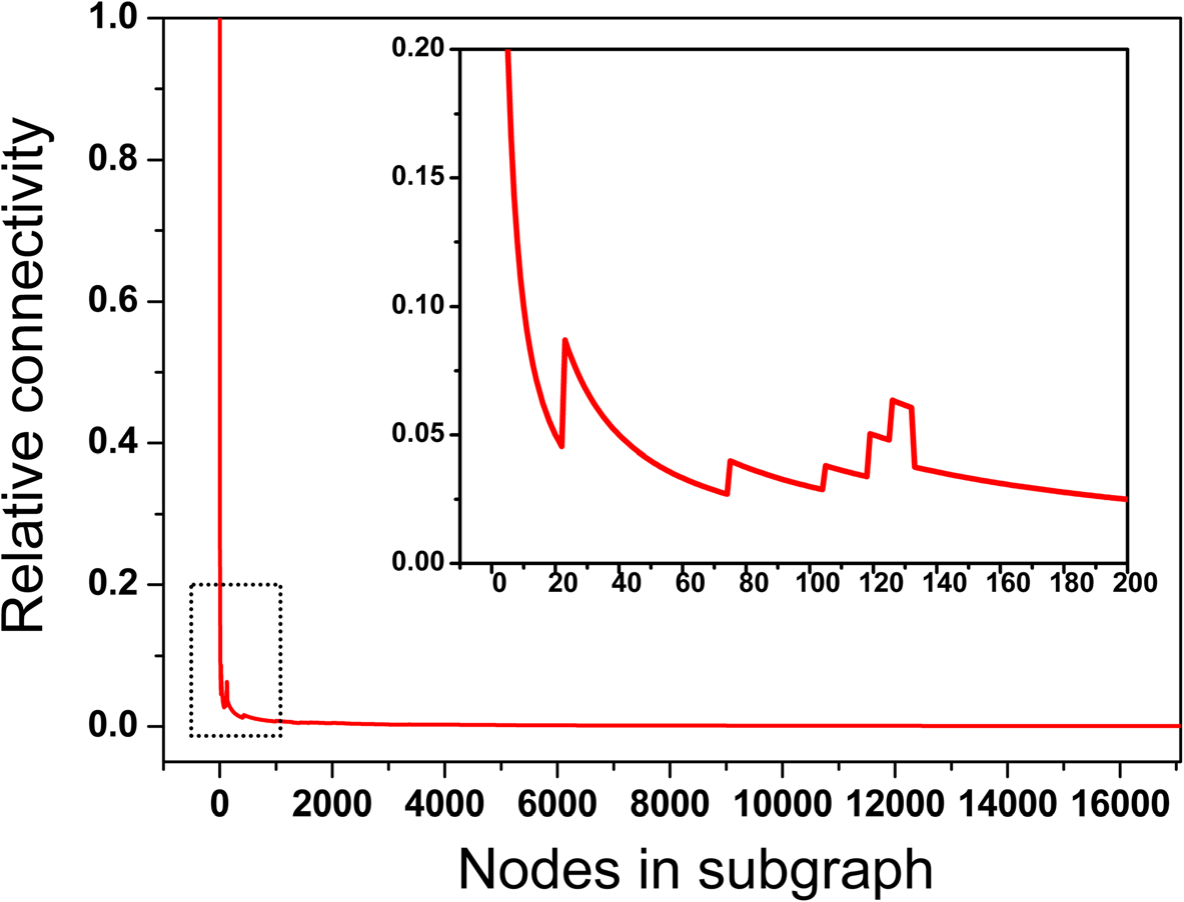
Relative subgraph connctivity as a funciton of number of nodes in the background network. The panel in this figure shows the change of relative connectivity in the node range between 1 and 200

The relative connectivity of subgraphs was computed as a function of node number and shown in Fig. 1. From this figure, we find that the relative connectivity is continual decrease when the number of nodes is less than 20. Subsequently, the relative connectivity shows some fluctuations with increase of nodes. When the number of nodes is greater than 132, the variation of relative connectivity becomes stable and reaches the relative connectivity of entire network. Therefore, we define the top 132 proteins in the degree ranking as the hub proteins in the background network. The Uniprot ID and name of each hub proteins is listed in Additional file 1: Table S1.

**Fig. 2:**
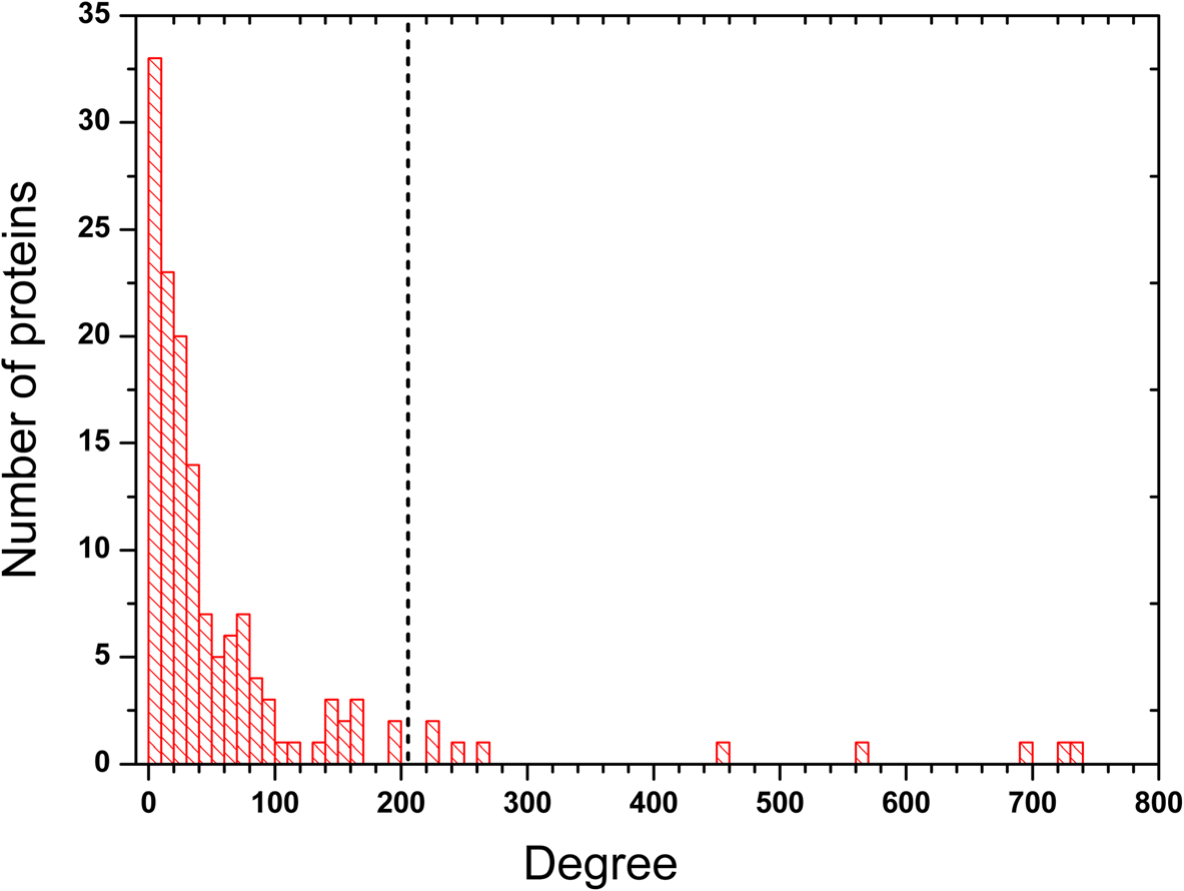
Degree distribution of HL-specific proteins in the background network. The black line in the figure is the lowest degree for hub proteins in the backgournd network. The HL-specific proteins whose degree is bigger than the lowest degree can be thought as the hub proteins in the background network. There are 10 HL-speicific proteins as the hub proteins in the background network

The degree distribution of HL-specific proteins in the background network is shown in Fig. 2. From this distribution we can find that 85% of HL-specific proteins have the degree with less than 100. According to the definition of hub proteins in the background network, only 10 HL-specific proteins belong to the hub proteins in the background network. Based on the guilt-by-association principle, we assume that the HL-specific proteins may be closely connected together in the background network. Whereas the 10 hub proteins might play an important role in connecting other HL-specific proteins. Therefore, we obtained a small network only consisting of HL-specific proteins from the background network. This small network is referred as HL-basic network.

#### HL-basic PPI network

**Fig. 3:**
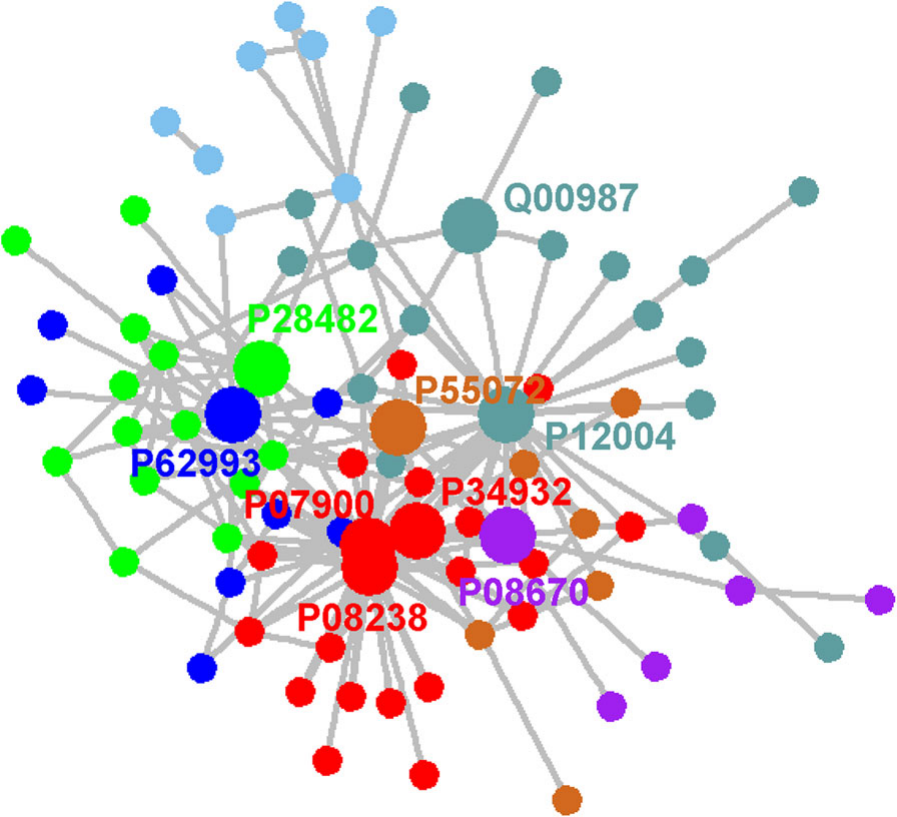
The sub-network consisted of 84 HL-specific proteins. The sub-network can be divided into eight modules in which the nodes are colored into different colors. 9 hub proteins are located into the different modules and shown in the bigger circle. Their Uniprot ID are also shown with the corresponding colors

In the HL-basic network, these are only 144 nodes and 180 edges. The nodes represent the HL-specific proteins and the edge is the interaction between two HL-specific proteins. The HL-basic network is displayed in Fig. 3. Based on the connection between nodes, 144 nodes can be distinctly classified into two groups. In one group, 84 out of 144 nodes are connected to form a sub-network and 9 hub proteins in the background network are included into this sub-network. The nodes in another group have not any interacting partners in HL-basic network. Moreover, according to the calculated maximum modularity score, the sub-network can be further divided into eight modules and 9 hub proteins are located respectively into different modules that are displayed in different colors in Fig. 3.

**Fig. 4:**
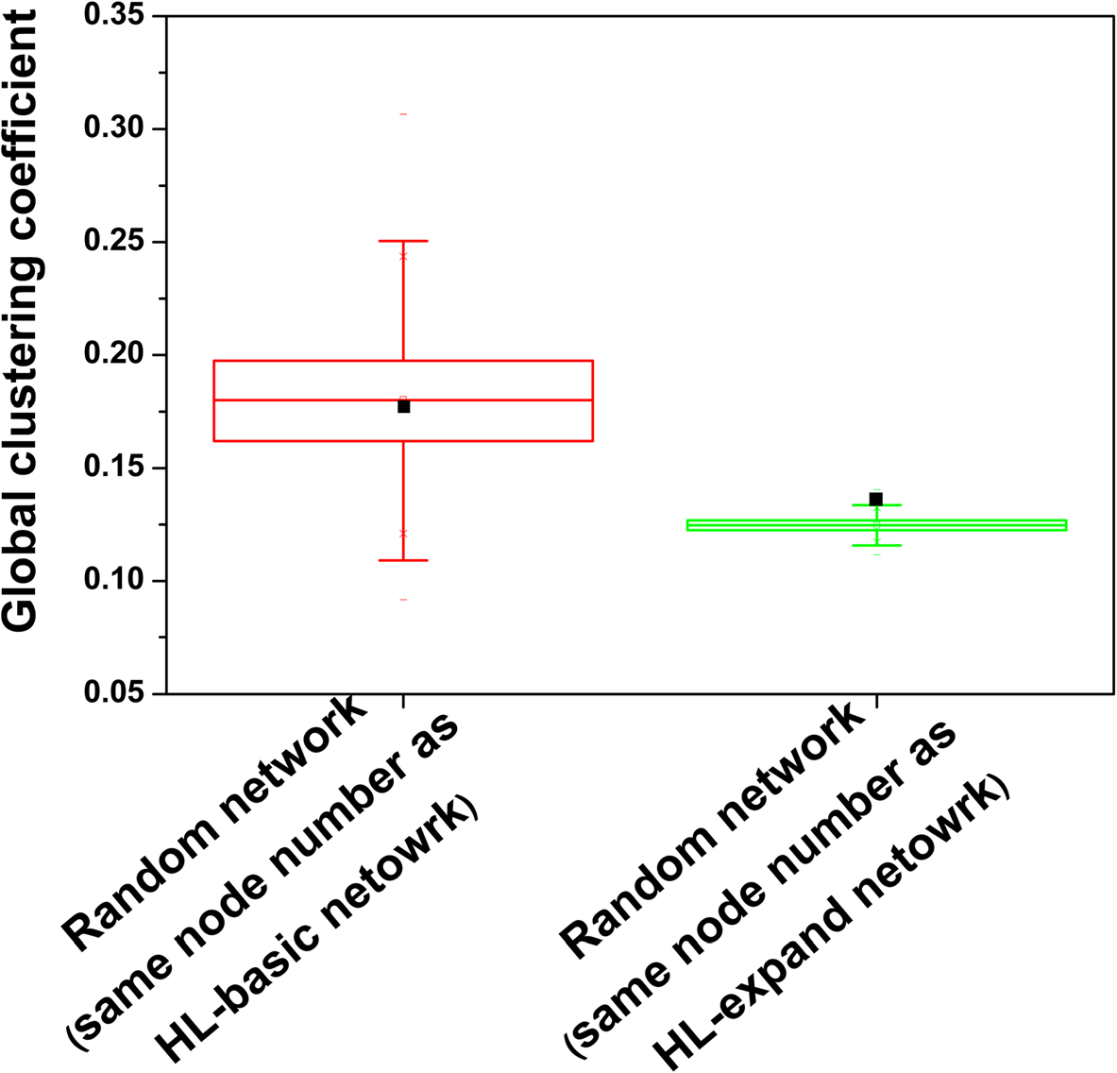
Comparison of global clustering coefficients of HL-basic network and HL-expand network with their corresponding random networks. The box plot displays the distribution of global clustering coefficients of 10,000 random networks that have the same numbers of nodes with equal degree as the HL-basic network and HL-expanded network, respectively. The black rectangle represents the global cluster coefficient of the HL-basic network or the HL-expanded network

Clustering coefficient is a measure of node aggregation in a network. We calculate the global clustering coefficient of the sub-network to evaluate the connection extent of the HL-specific proteins. The global clustering coefficient is calculated to be 0.17. To confirm whether that the HL-associate proteins are more closely connected together, we generated 10,000 random networks consisted of the same number of nodes as the sub-network. Subsequently, we also calculated the global clustering coefficients of random networks and compared them with that of the HL-basic network. The comparison results are shown in Fig. 4. It can be seen that the global clustering coefficient of HL-basic network lies within the same range as those of 10,000 random networks. The result indicates that the HL-associate proteins are not so densely connected together compared with the randomly selected proteins. According to Local hypothesis that proteins involved in the same disease tend to interact with each other [1], it implies that in this study the list of collected HL-specific proteins is not entirely comprehensive. Moreover, 60 isolated nodes in the HL-basic network also confirm this observation.

**Fig. 5:**
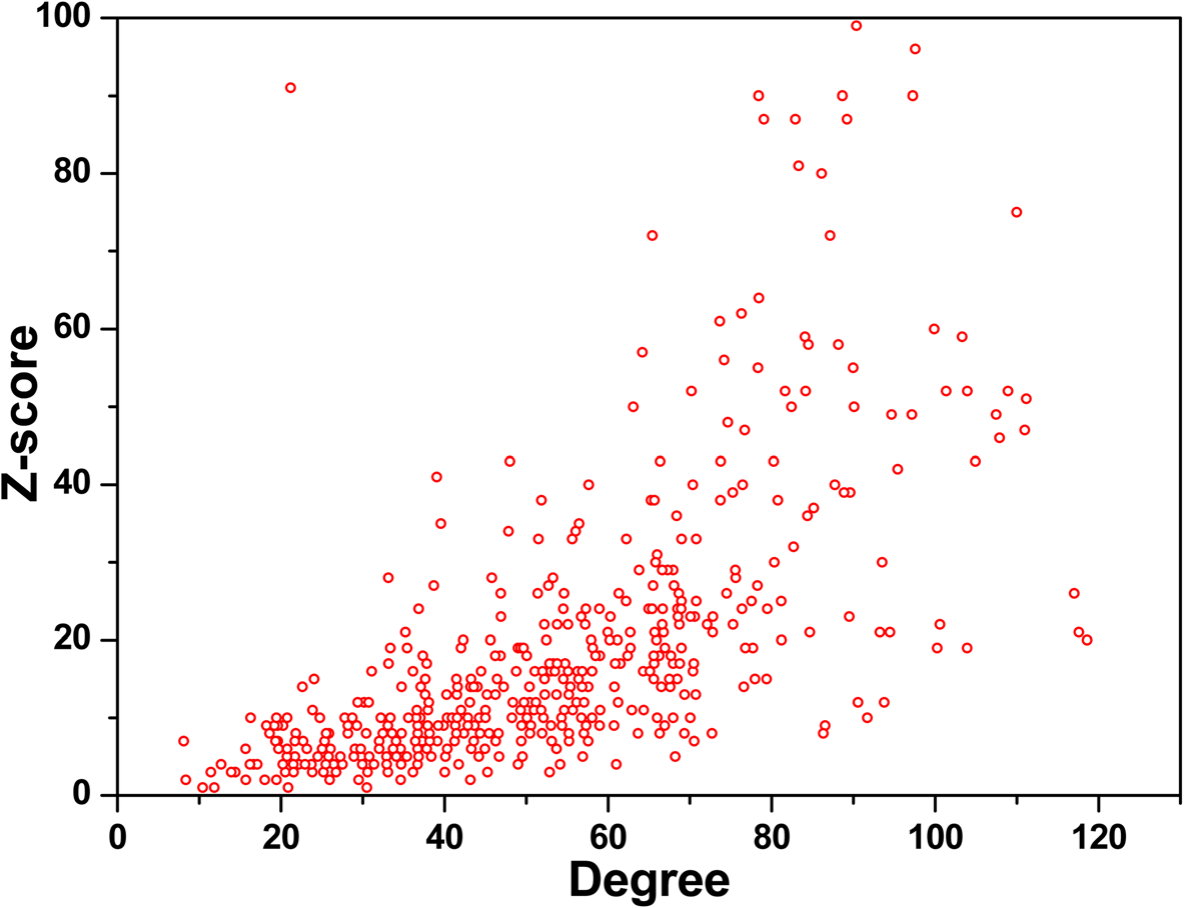
Z-score distribution of 541 nodes in HL-expanded network along with their degree values

#### HL-expanded network

On the basis of the above results, we think the HL-basic network is yet incomplete. In order to construct a more comprehensive HL-related network, we regarded the 144 HL-specific proteins as seed proteins and then selected their neighbors that directly connected with them in the background network. The newly selected proteins and the involving interactions were integrated to build a network called as HL-expanded network. This resulting network comprises 541 nodes and 5057 connections.

Compared with the HL-basic network, the HL-expanded network contains more hub proteins. There are a total of 61 hub proteins identified from the background network. These hub proteins make the nodes in the HL-expanded network densely connect to each other. Similarly, we also generated 10,000 random networks where nodes have the same degree distribution as those of the HL-expanded network and compared the global clustering coefficient between HL-expanded network and random networks. The global clustering coefficient of HL-expanded network is computed to be 0.135, which is higher the average value of 10,000 random networks (0.124) as shown in Fig. 4. The statistics analysis using Kolmogorov-Smirnov test (*p*-value =2.2 × 10^− 16^) also validates the observation that the global clustering coefficient of HL-expanded network significantly differs from those derived from the random networks. It indicates that, as expected, the HL-specific proteins are densely connected together.

In addition, as components of the background network, the nodes in the HL-expanded network simultaneously connect with other nodes out of the HL-expanded network. To evaluate the extent of connection between the nodes inside and outside the HL-expanded network, we calculate Z-score value that is based on the degree values in the HL-expanded network and the background network. If the Z-score of a node is larger than 0, it means this node has more interaction with the nodes within the HL-expanded network. On the contrary, the node is more connected with the nodes in the background network.

Figure 5 displays the Z-score distribution of all nodes in the HL-expanded network with their degree values. From this figure, we can clearly find that Z-scores of all nodes are basically correlated with their degree. Moreover, the Z-scores of all nodes in the HL-expanded network are larger than 0, meaning that all nodes in HL-expanded network tend to connect with the intra-network nodes and form a relatively isolated network from the background network.

In summary, the HL-expanded network is a relatively compact network, in which 144 HL-associate proteins are tightly linked together. Based on Local hypothesis that proteins involved in the same disease tend to interact with each other, the HL-expanded network can be considered as HL-specific network and all proteins in this network are regarded to be related to HL.

The above results display that the HL-expanded network possesses higher cluster coefficient compared with the random network. It suggests that the HL-expanded network may be a small-world network. Hence we adopted a measurement of S^△^ index proposed by Humphries and Gurney [31] to quantify the small-worldness of this network. The calculated S^△^ value is 4.55, greater than 1. It means that HL-expanded network is a small world network. Because the small-world network tends to contain cliques, we further perform clustering analysis for the HL-expanded network.

**Fig. 6:**
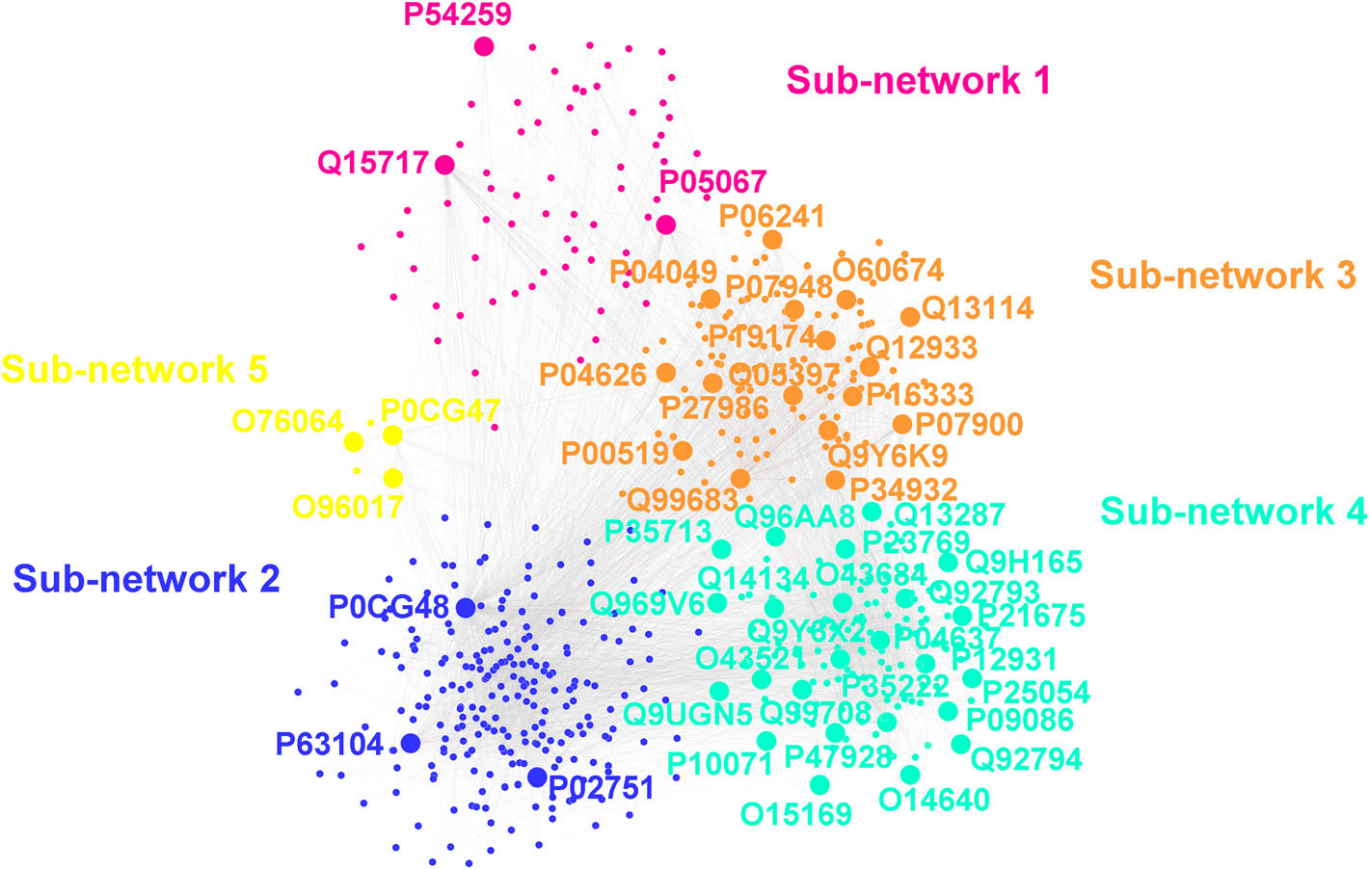
The HL-expanded network and its constitued sub-networks. Based on dense connections of nodes, the HL-expanded network is divided into 5 sub-networks. The nodes within each sub-network are colored in different colors. The nodes with bigger size are the hub proteins in each sub-network and are labeled with Uniprot ID

The results of cluster analysis show that the HL-expanded network can be divided into five sub-networks, in which the nodes have a high tendency to connect with each other. The HL-expanded network and its sub-networks are shown in Fig. 6. Subsequently, we conducted functional enrichment analysis and KEGG pathway analysis on five sub-networks respectively.

The enrichment results are listed in Additional file 1: Table S2. From this table, we can observe that five sub-networks are separately involved in the different functions and pathway. For example, the proteins in sub-network 2 mainly participate in the process of cell-cell adhesion, which may be related to the migration of lymphoma cells. Meanwhile, the proteins in this sub-network are also involved in pathway of Epstein-Barr virus infection, which has been confirmed to be an important cause for HL [32]. In addition, the proteins contained in sub-network 3 are associated with kinase activity and signaling pathway, particularly NF-kappa B signaling pathway. The aberrant NF-kappa B activity has been recognized as a critical pathogenic factor in lymphoma [33]. Moreover, the pathway enrichment results also shown that the proteins in Sub-network 4 are participated in the process of human T-lymphotropic virus I (HTLV-I) infection and colorectal cancer. This is consistent with the fact that HILV-I infection is the cause of adult T-cell lymphoma [34] and colorectal cancer is a common secondary cancer in HL survivors [35]. These results directly validate the rationality of the constructed HL-expanded network.

Because in the network the clusters is generally formed by the high-connectivity hubs proteins [36], we can further simplify the network to the connection of hub proteins. By extracting the hub proteins in five sub-networks and their mutual connections, we build a simplest form of HL-expanded network. This simplified network consists of 49 nodes shown in the Fig. 6, which can directly connect 470 out of remaining 492 nodes in HL-expanded network and play an important role in maintaining the HL-expanded network structure. Therefore, these nodes can be considered to make up the backbone of HL-expanded network and the corresponding proteins represented by these nodes are considered as the key proteins for HL. The Uniprot ID numbers of 49 proteins together with their name and possible functions in HL are listed in Additional file 1: Table S3.

Among 49 key proteins, 18 proteins are the manually collected HL-related proteins and 4 proteins, P54529, P04637, Q13287 and P12931 have also been proven to be related with the development of HL. The result directly confirmed the correctness of the identification of key proteins based on the context of network. Remaining 27 proteins as the candidates can be further studied using the experimental methods. Meanwhile all 49 key proteins can also be regarded as the potential targets for treatment of HL.

### Prediction of miRNA targets

In addition to the related proteins, many studies have confirmed that miRNAs are closely associated with the HL. Some specific miRNAs can be used to differentiate HL lymph nodes from reactive lymph nodes and HRS cells from germinal center B cells [37]. They are also utilized to track treatment response for HL [38]. However, regarding how miRNAs participate in the development of HL and regulate the interaction between HL-specific proteins, it is not completely clear. Hence, we further obtained the regulatory relationships between miRNAs and HL-specific proteins from two miRNAs target databases and analyzed the regulations of miRNAs on protein interaction network. In this study, we extracted a total of 14,614 and 14,693 experimentally validated miRNA-target interactions from miRWalk and miRTarbase, respectively. The intersection of two datasets is retained for further analysis.

**Fig. 7:**
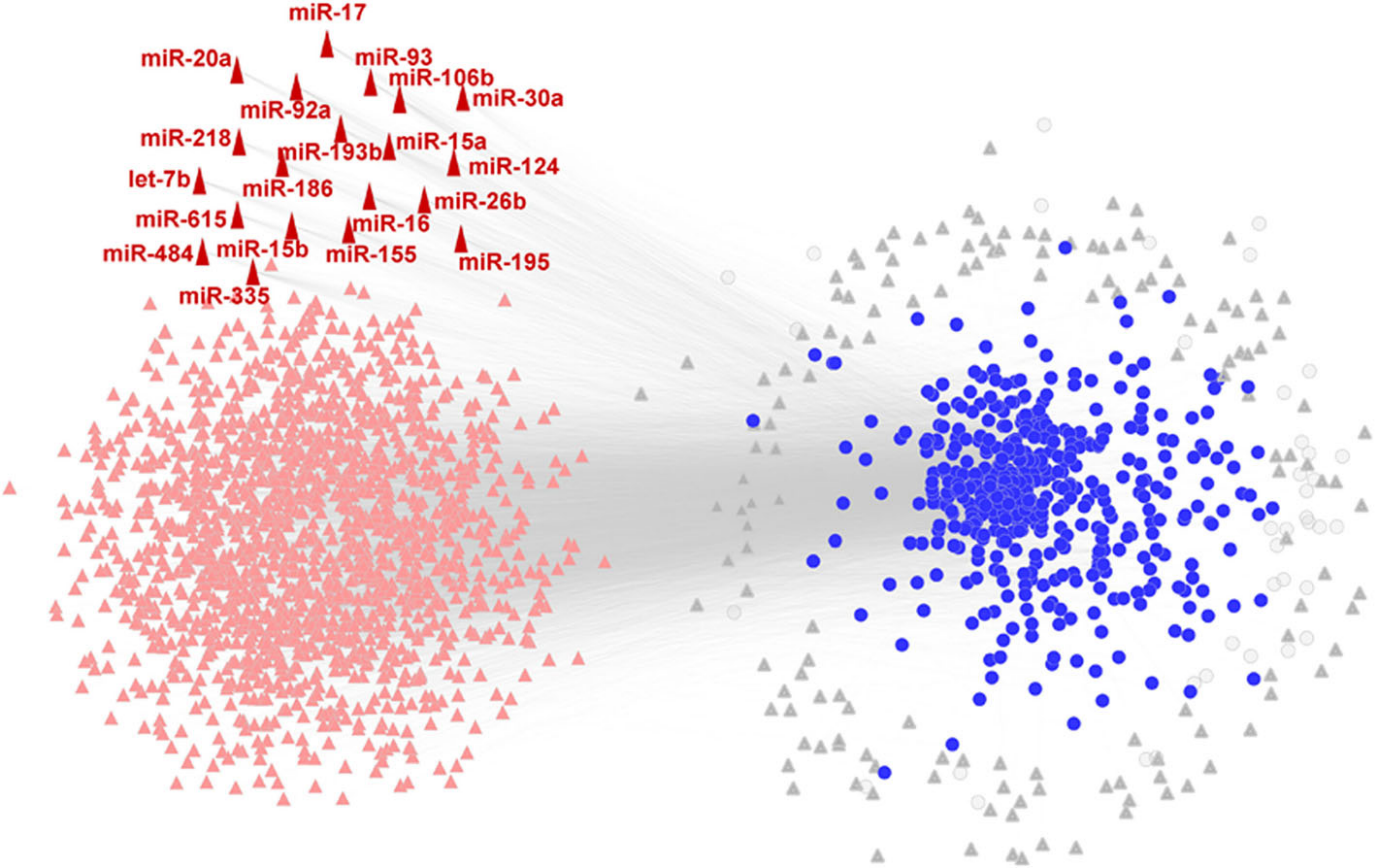
The HL-specific miRNA-protein network consisted of HL-specific proteins and miRNAs. 497 HL-specific proteins are depicted as black circles and 1628 miRNAs are shown in pink triangle. But the HL-proteins that are regulated only by one miRNA and miRNAs that only modulate one HL-protein are colored by gray. 20 key miRNAs are colord as red and their name are labeled

Based on the obtained experimentally validated miRNA-target data, we construct a HL-specific miRNA-protein network (shown in Fig. 7), in which there are 497 HL-specific proteins and 1628 miRNAs as well as 14,299 miRNA-protein interactions. Although, in this network, 40 HL-specific proteins are regulated only by one miRNA and 152 miRNAs modulate one protein, most of miRNAs and proteins have many-to-many regulatory relations. Among the 1628 miRNAs, 20 miRNAs can directly regulate approximately 80% of proteins in this network. So these 20 miRNAs can be considered as key miRNAs for HL and 3 out of 20 miRNAs are included in the previously identified HL-related miRNAs.

### Analysis of miRNAs regulation from the network perspective

**Fig. 8:**
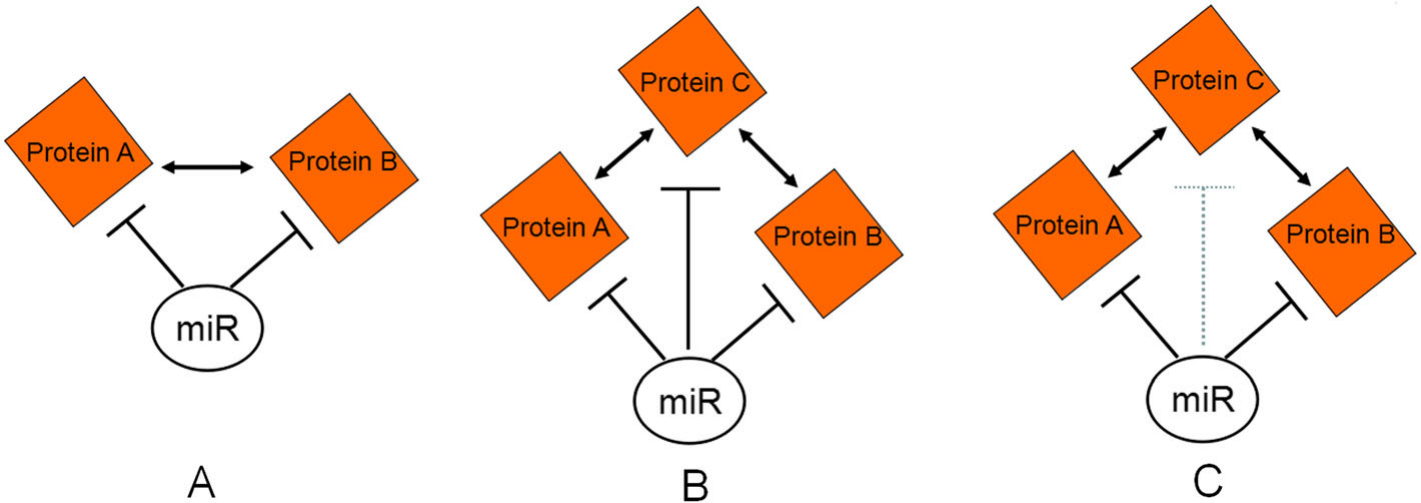
Illustration of three types of miRNA regulations on HL-specific network. **a** A miRNA simultaneously regulates two interacting proteins. **b** A miRNA mediates three sequentially interacting proteins. **c**. A miRNA directly regulates two out of three sequentially interacting proteins and indirectly mediates one out of three sequentially interacting proteins

It has been demonstrated that the targets of miRNAs are generally more connected in the protein-protein interaction network than expected by chance [18, 39]. The protein-protein interaction may enhance regulatory effect of miRNAs on targets. Therefore, we integrate protein-protein interactions with miRNA-protein regulation to explore the miRNAs-mediated regulation on the protein network. In this study, we will consider three simplest types of regulatory patterns. The first pattern is that a miRNA can simultaneously regulate two interacting proteins shown in Fig. 8a. By means of the interaction between two proteins, miRNA may strengthen the regulatory effect on them. In the HL-expanded network, out of total 5057 interacting protein pairs, 2336 pairs are regulated in this way. This result demonstrates this kind of regulation is a common pattern in the HL-expanded network and it is agreement with the previous study [39]. If taking account into this type of regulation, 20 key miRNAs can not only target 80% of HL-specific proteins, but also regulate approximately 60% of interacting protein pairs in the HL-expanded network. Hence 20 key miRNAs are playing an important role in regulating HL-specific proteins and network.

Besides regulation of the interacting protein pairs, we further analyze the regulatory pattern that three sequentially interacting proteins are mediated by a miRNA (shown in Fig. 8b). Compared with the first type of pattern, this pattern can more efficiently strengthen the regulatory effect of miRNA through combination of double protein-protein interactions. In the case of HL-specific proteins, total 341 proteins are found to be mediated in this way by 550 miRNAs, and 20 key miRNAs are found to regulate up to 54% of all HL-specific proteins.

The third type of regulation pattern is similar with the second one and is also involved in mediating three sequentially interacting proteins (shown in Fig. 8c). But differing from the second pattern, the protein that interacts with other two proteins is not a target of the miRNA, but it may be indirectly regulated by this miRNA through mediating two interacting proteins. It means that by means of protein interactions, miRNA not only enhance the regulatory effect, but also expand the regulatory scope.

Thus, when we only consider the directly regulation of miRNAs, 45 miRNAs can regulate approximately 90% of proteins in the HL-expand network. On the contrary, when all three types of regulations are taken into account, only 5 miRNAs are able to regulate the same number of proteins. Therefore, the 5 miRNAs are thought to be the core miRNAs that they can regulate almost all HL-related proteins in the HL-expanded network. Moreover, the 5 core miRNAs also rank in the top 5 among 20 key miRNAs identified above.

### Construct a simplified network consisting of core miRNAs and key proteins

**Fig. 9:**
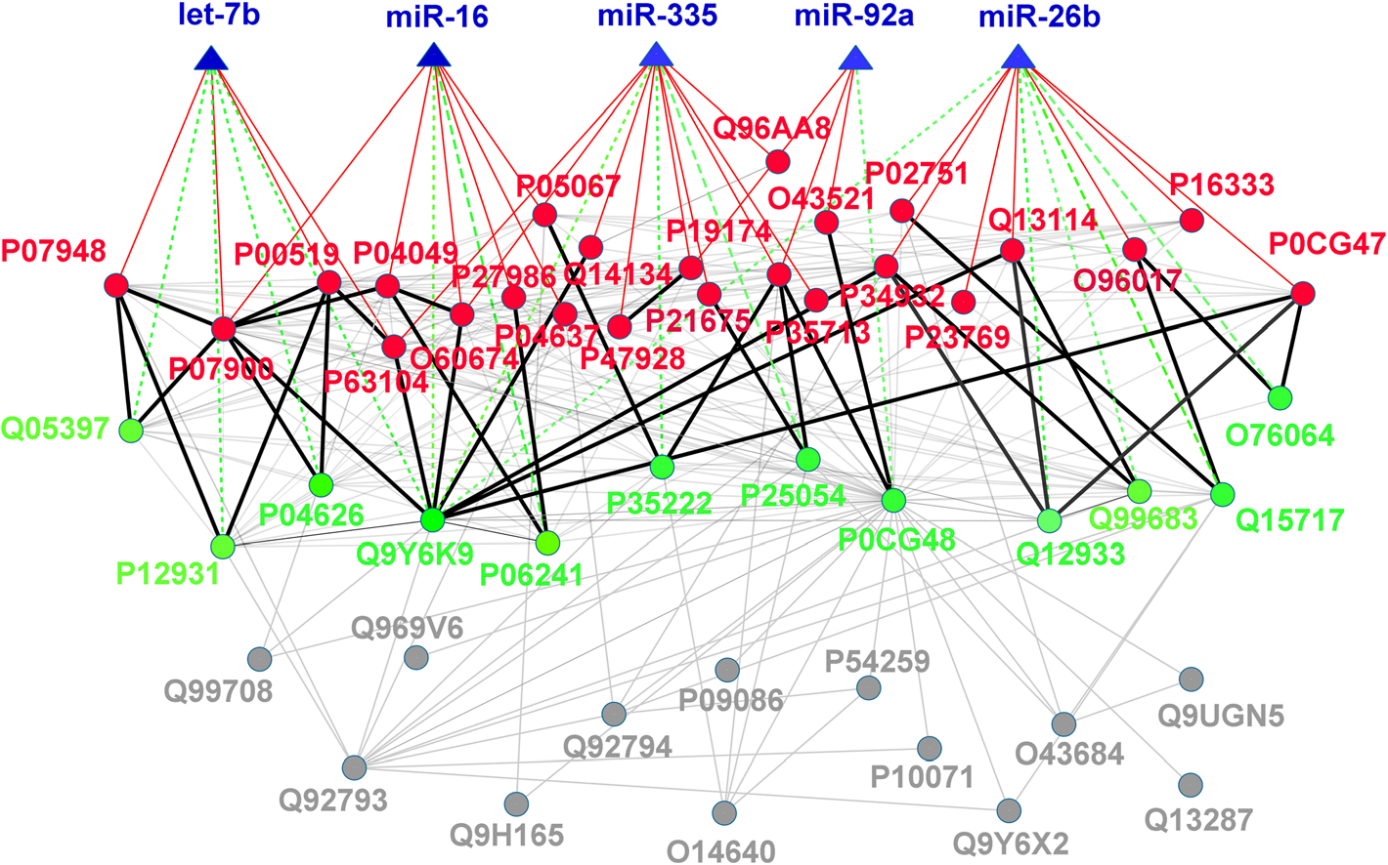
Simplified miRNA-regulated HL-specific protein network consisting of 5 core miRNAs and 49 key HL-specific proteins. The core miRNAs are shown in blue rectangle and their name are also labeled in blue. The HL-specific proteins are shown in circle with different colors. The proteins in red color can be directly regulated by 5 core miRNAs. The direct miRNA regulation on protein is also shown in solid red line. The proteins in green color are indirectly regulated by the means of two interacting proteins. These proteins interaction is shown in solid black line. The proteins in gray color can’t be regulated by the core miRNAs in direct and indirect ways

Based on the miRNA regulation on the protein network, we identified 5 core miRNAs from 1628 miRNAs. To better understand relation between miRNAs and HL-specific proteins, we construct a simplified network only consisting of 5 core miRNAs and 49 key proteins. Fig. 9 displays this network where the edges represent two types of information, miRNA-protein regulation and protein-protein interaction.

As the main backbone of the HL-expanded network, the 49 key proteins are highly important for maintaining structural integrity of network. Therefore, by targeting the 49 key proteins, the 5 core miRNAs are nearly able to regulate the entire HL-expanded network. In terms of influence on the network, the 5 core miRNAs are thought to be closely related with HL. Three out of five miRNAs, miR-92a, miR-26b and let-7b, are specifically expressed in Hodgkin lymphoma cell line [40] [41] and the remaining two miRNAs, miR-335 and miR-16, have identified to be breast cancer [42] and acute myelogenous leukemia (AML) [43]. However, it is not entirely clear how these 5 core miRNAs are involved in HL pathology. Because the function of miRNAs may be determined by regulating the function of their targeting proteins [44, 45], we explore the role of 5 core miRNAs in HL based on the function of key proteins. Additional file 1: Table S4 lists the proteins regulated directly and indirectly by 5 core miRNAs and their possible functions in HL derived from their the functions of their targets. Among 49 key proteins, 24 proteins are directly regulated by all 5 core miRNAs. The pathway enrichment was performed using these 24 key proteins and several pathways were found to be associated with these proteins, including ErbB signaling pathway, Focal adhesion, Viral carcinogenesis, Sphingolipid signaling pathway, VEGF signaling pathway and Epstein-Barr virus infection. It implies that the 5 core miRNAs may be associatd with HL by regulating these pathways. According to the enrichment results, virus infection especially Epstein-Bar virus infection may contribute to the development of HL, which has been discussed in details elsewhere [46]. In addition, most of key proteins are enriched in four signaling pathways associated with cancer development and progression, suggesting that HL may not be related with a single or unique pathway and the abnormalities of several pathways may cause the occurrence and development of HL.

## Discussion

Currently, the application of high-throughput techniques in HL generated a larger amount of data. Based on these data, many HL-related proteins and miRNAs have also been identified. But it remains thoroughly unclear how these HL-related molecules participate in the pathology of HL and how the HL-related miRNAs regulate the HL-related proteins and their constituted PPI network. These information may help to search for key proteins and miRNAs that can be considered as biomarkers and drug targets for HL. The purpose of this study is to obtain important proteins and miRNAs and to reveal their regulatory relationship under the scale of network.

In this study, we constructed a series of network models. Initially, we built three different but related PPI networks. By analyzing those three networks, we investigate the connection characteristic of the HL-related proteins and find that these proteins are prone to connect with each other compared with other proteins. Subsequently, we obtained a PPI network closely associated with HL and 49 key proteins. These key proteins play imperative role in maintaining the integrity of the HL-related PPI network. Hence these key proteins have a higher probability to involve into initial and development of HL. They can be further studied for being the reliable biomarkers and drug targets for HL using the experimental methods.

In addition, we also investigated the miRNA regulation on HL-related PPI network and analyzed three kinds of simple regulation patterns. Based on these regulations on HL-related PPI network, we identified 5 core miRNAs that can mediate approximately 90% of proteins in the HL-related PPI network. When the expression of these 5 miRNAs is altered, the proteins in this network can be to some extent influenced by the regulation of these miRNA, which may cause the occurrence of HL. Therefore, these 5 miRNAs can be considered as the potential biomarkers for the diagnosis of HL.

To better understand the relation between 49 key proteins and 5 core miRNAs, we finally constructed a PPI network combined with the regulation of miRNAs. This network indicates that it is necessary for comprehensive understanding the regulation of miRNAs on targets to fully take into account of the related protein interactions. Based on the analysis of this combined network, we identified several protein pathways closely associated with HL, including ErbB signaling pathway, Focal adhesion, Viral carcinogenesis, Sphingolipid signaling pathway, VEGF signaling pathway and Epstein-Barr virus infection. These information will be helpful to elucidate HL mechanisms and identify pharmaceutical targets.

## Conclusion

In this study, we use a three-step strategy to construct a HL-specific network that is as complete as possible. Firstly we constructed a background protein-protein interaction network based on the current PPI information. According to the background network, we then build a HL-basic network only consisting of the HL-associated proteins. Finally, we obtained a complete HL-specific protein-protein network. The HL-specific network consists of 541 proteins and 5057 protein interactions. Moreover, the HL-specific network is further divided into five sub-networks and 49 proteins are identified as the important nodes that make up and connect these 5 sub-networks. Therefore, we consider the 49 proteins as the key proteins of HL.

In addition, based on the experimentally validated information about miRNA-target, we get the regulatory relation between miRNAs and HL-specific network. Furthermore, we investigate three simple regulatory patterns of miRNA in the HL-specific network, The co-regulation of protein pairs is the main regulatory pattern of miRNAs on the protein network in the HL-specific network.

Finally, we identified 5 core miRNAs and 49 key proteins from the point of view of network. These molecules can be thought as the potential biomarker in the diagnosis of HL. Their mutual regulatory interactions provide a foundation for further studying the mechanism of HL and identifying the potential drug targets for treatment of HL.

## Methods

### Collection of HL-related proteins and miRNAs

The proteins associated with HL were obtained by collecting experimental data from published studies and searching public databases. The experimental data mainly came from two high-throughput proteomics-based studies that aimed to identify proteins specifically expressed in HL-derived cells [24, 47]. A total of 120 proteins were identified to be highly associated with HL. In order to obtain more HL-associated proteins, we further conducted a general database search on two databases, i.e., NCBI and Uniprot, using “Hodgkin lymphoma and *Homo sapiens*” as query keywords. Altogether, 92 proteins were retained after filtering out duplicate entries. After gathering all proteins and removing duplicate ones, we finally obtained 178 HL-associated proteins for subsequent network analysis.

HL-associated miRNAs were also obtained from the specific experimental data and related database. Based on a miRNA microarray analysis, 77 miRNAs exclusively expressed in Hdgkin and Reed Sternberg cells were extracted and considered to be relevant to HL. In addition, a group of HL-associated miRNAs was obtained from dbDEMC [48], a database of differentially expressed miRNA in human cancers. Finally, a total of 121 miRNAs were included for subsequent analysis.

### Construction of PPI networks

In this study, we used a three-step strategy to construct a comprehensive and reliable protein interactions network related to HL from the collected HL-associated proteins. Firstly, we built a background protein interactions network that includes as many proteins as possible. The protein-protein interaction (PPI) data for the network was mainly extracted from five primary PPI databases, DIP [49], MINT [50], IntAct [51], BioGrid [52] and HPRD [53]. Only the experimentally validated PPI, such as physical interactions (MI:0218), direct interactions (MI:0407) and physical associations (MI:0915), are selected from these databases. Additional file 1: Table S5 lists the respective number of PPI data from five databases. All extracted PPI data were merged together and duplicate data were deleted. A total of 146,295 PPI data involving 17,076 proteins were retained to construct the PPI background network.

Next, we chose the PPI data involving HL-associated proteins from the background network and built a small network only consisting of the HL-associated proteins. This small network can be considered as a HL-basic network. Finally, based on the “guilt by association” principle that two interacting proteins in a PPI network might also share a function or involve the same disease [54, 55], we took the HL-associated protein in the HL-basic network as seed protein and select their all connected nodes in the background network to construct a expanded PPI network. This resulting network could be considered as a comprehensive and reliable network specific to HL for further analysis.

### Identification of hub proteins

In this study, we applied the method proposed by Raval et al. [29] to identify the hub proteins in PPI network. This method is just based on the topology of network. Firstly, all nodes in the PPI network were ranked in decreasing order of degree. Subsequently, a succession of subgraphs was generated by successively adding nodes in descending order of degree. The relative connectivity of each subgraph was calculated as the number of nodes in the largest component of a subgraph divided by the total number of nodes in this subgraph. Because the interactions between hubs are suppressed in the network [30], the connectivity of subgraphs consisting of hub proteins is relatively small. With the addition of no-hub proteins into the subgraph, its relative connectivity becomes gradually larger. Therefore, when the connectivity of subgraphs begins to rise and eventually reaches the connectivity of the entire network, the nodes included in this subgraph could be considered as the hub proteins.

### Generation of random networks

From the protein background network, we randomly selected the nodes that had the same degree distribution as the network of interest. Moreover, we also extracted the interaction between the selected nodes. Ultimately, based on these nodes and their interaction, the random network was generated. Compared with the network of interest, the random network does not have any biological meaning.

### Calculation of Z-score

In order to quantitatively evaluate the connection extent of the nodes with the nodes in two respective networks, we calculate Z-score of node using the binomial proportion test as follows:

$$ z=\frac{\left(\frac{a}{c}-\frac{b}{d}\right)}{\sqrt{\frac{\frac{b}{d}\left(1-\frac{b}{d}\right)}{d}}} $$

where a is the links of node in a network, c represents the total links in this network. Similarly, b equals the links of this node in another network and d is the total links in this network. If the Z-score of node is larger than 0, it indicates this node is more highly connected with the nodes in one network than another network and vice versa.

### Analysis of PPI network

The igraph package is used to calculate clustering coefficient of network, evaluate the small-worldness and perform modular analysis. GO and pathway enrichment analysis are conducted using the package clusterProfiler. All packages used in this study are run in R environment 3.3.2. The visualization of network is performed using Cytoscape Version 3.2.1.

### Identification of miRNAs regulating HL-specific proteins

Two major databases, miRTarBase [56] and miRWalk [57], are used to obtain miRNA-target interactions. Firstly, we extracted all experimentally validated miRNA-target interactions of *Homo sapiens* from two databases, respectively. Subsequently, we only selected the interactions involving the HL-specific proteins based on the gene name. Finally, the intersection between two data sets is retained for further analysis.

## Additional file

Additional file 1: **Table S1.** Uniprot ID and protein name for 132 hub proteins in Background network. **Table S2**:The enrichment results of five sub-networks in the HL-expanded network. **Table S3.** Uniprot ID of 49 key proteins and their related information in the HL-extended network.**Table S4.** Proteins mediated directly and indirectly by five core miRNAs and the possible functions of miRNA in HL. **Table S5.** Number of PPI data extracted from five databases and the database version. **Figure S1.** Degree distribution of PPI background network. (DOC 336 kb)

## Data Availability

The datasets used and/or analysed during the current study are available from the corresponding authors on reasonable request.

## Abbreviations

AML: Acute myelogenous leukemia
HL: Hodgkin Lymphoma
HTLV-I: Human T-lymphotropic virus I
miRNA: microRNA
PPI: Protein-protein interaction
