# Supplementary material for: Analyzing the regulation of miRNAs on protein-protein interaction network in Hodgkin lymphoma
Source: BMC Bioinformatics. 2019 Sep 2;20:449. doi: 10.1186/s12859-019-3041-9 (PMC6720096; doi:10.1186/s12859-019-3041-9)
Supplement: Supplementary file 1 — Table S1. Uniprot ID and protein name for 132 hub proteins in Background network. Table S2:The enrichment results of five sub-networks in the HL-expanded network. Table S3. Uniprot ID of 49 key proteins and their related information in the HL-extended network.Table S4. Proteins mediated directly and indirectly by five core miRNAs and the possible functions of miRNA in HL. Table S5. Number of PPI data extracted from five databases and the database version. Figure S1. Degree distribution of PPI background network. (DOC 336 kb) [file 12859_2019_3041_MOESM1_ESM.doc]

Table S1: Uniprot ID and protein name for 132 hub proteins in Background network

| Uniprot | Protein Name | Uniprot | Protein Name |
| --- | --- | --- | --- |
| P0CG48 | Polyubiquitin-C | Q7L5N1 | COP9 signalosome complex subunit 6 |
| P05067 | Amyloid-beta precursor protein | P63279 | SUMO-conjugating enzyme UBC9 |
| Q15717 | ELAV-like protein 1 | P54274 | Telomeric repeat-binding factor 1 |
| P61956 | Small ubiquitin-related modifier 2 | Q9HCE7 | E3 ubiquitin-protein ligase SMURF1 |
| P63165 | Small ubiquitin-related modifier 1 | Q13620 | Cullin-4B |
| Q13618 | Cullin-3 | O95793 | Double-stranded RNA-binding protein Staufen homolog 1 |
| P02751 | Fibronectin | P11142 | Heat shock cognate 71 kDa protein |
| Q92905 | COP9 signalosome complex | Q96EB6 | NAD-dependent protein deacetylase sirtuin-1 |
| Q00987 | E3 ubiquitin-protein ligase Mdm2 | P27986 | Phosphatidylinositol 3-kinase regulatory subunit alpha |
| P07900 | Heat shock protein HSP 90-alpha | Q9P2P5 | E3 ubiquitin-protein ligase HECW2 |
| Q15843 | NEDD8 | O60341 | Lysine-specific histone demethylase 1A |
| Q99496 | E3 ubiquitin-protein ligase RING2 | P49841 | Glycogen synthase kinase-3 beta |
| P62993 | Growth factor receptor-bound protein 2 | P62136 | Serine/threonine-protein phosphatase PP1-alpha catalytic subunit |
| Q9NRC8 | NAD-dependent protein deacetylase sirtuin-7 | P10275 | Androgen receptor |
| P01106 | Myc proto-oncogene protein | P30480 | HLA class I histocompatibility antigen, B-42 alpha chain |
| O15205 | Ubiquitin D | P46108 | Adapter molecule crk |
| P04637 | Cellular tumor antigen p53 | Q13148 | TAR DNA-binding protein 43 |
| P63104 | 14-3-3 protein zeta/delta | P28482 | Mitogen-activated protein kinase 1 |
| Q13616 | Cullin-1 | Q12933 | TNF receptor-associated factor 2 |
| Q86VP6 | Cullin-associated NEDD8-dissociated protein 1 | P31749 | RAC-alpha serine/threonine-protein kinase |
| P03372 | Estrogen receptor | P46934 | E3 ubiquitin-protein ligase NEDD4 |
| Q9NRD1 | F-box only protein 6 | P08047 | Transcription factor Sp1 |
| P00533 | Epidermal growth factor receptor | O15379 | Histone deacetylase 3 |
| P24941 | Cyclin-dependent kinase 2 | P49407 | Beta-arrestin-1 |
| P08238 | Heat shock protein HSP 90-beta | Q6ZW49 | PAX-interacting protein 1 |
| Q13547 | Histone deacetylase 1 | P11441 | Ubiquitin-like protein 4A |
| Q9Y4K3 | TNF receptor-associated factor 6 | Q04206 | Transcription factor p65 |
| P13612 | Integrin alpha-4 | Q13619 | Cullin-4A |
| O75530 | Polycomb protein EED | P07550 | Beta-2 adrenergic receptor |
| P55072 | Transitional endoplasmic reticulum ATPase | P34932 | Heat shock 70 kDa protein 4 |
| Q09472 | Histone acetyltransferase p300 | P42858 | Huntingtin |
| P27694 | Replication protein A 70 kDa DNA-binding subunit | P67870 | Casein kinase II subunit beta |
| P19320 | Vascular cell adhesion protein 1 | Q99728 | BRCA1-associated RING domain protein 1 |
| P12931 | Proto-oncogene tyrosine-protein kinase Src | Q15910 | Histone-lysine N-methyltransferase EZH2 |
| Q9Y6K9 | NF-kappa-B essential modulator | Q15554 | Telomeric repeat-binding factor 2 |
| P38398 | Breast cancer type 1 susceptibility protein | P16333 | Cytoplasmic protein NCK1 |
| P68400 | Casein kinase II subunit alpha | Q9NZC7 | WW domain-containing oxidoreductase |
| P40337 | von Hippel-Lindau disease tumor suppressor | P17252 | Protein kinase C alpha type |
| O95817 | BAG family molecular chaperone regulator 3 | P22681 | E3 ubiquitin-protein ligase CBL |
| P61981 | 14-3-3 protein gamma | P23508 | Colorectal mutant cancer protein |
| P15927 | Replication protein A 32 kDa subunit | P09651 | Heterogeneous nuclear ribonucleoprotein A1 |
| Q13617 | Cullin-2 | P29353 | SHC-transforming protein 1 |
| P27348 | 14-3-3 protein theta | P05412 | Transcription factor AP-1 |
| P84022 | Mothers against decapentaplegic homolog 3 | P06400 | Retinoblastoma-associated protein |
| Q15796 | Mothers against decapentaplegic homolog 2 | Q9UBN7 | Histone deacetylase 6 |
| O60260 | E3 ubiquitin-protein ligase parkin | P08670 | Vimentin |
| Q93034 | Cullin-5 | P12956 | X-ray repair cross-complementing protein 6 |
| Q92769 | Histone deacetylase 2 | Q13485 | Mothers against decapentaplegic homolog 4 |
| P06748 | Nucleophosmin | P17612 | cAMP-dependent protein kinase catalytic subunit alpha |
| P35244 | Replication protein A 14 kDa subunit | P12004 | Proliferating cell nuclear antigen |
| Q14164 | Inhibitor of nuclear factor kappa-B kinase subunit epsilon | P78362 | SRSF protein kinase 2 |
| P35226 | Polycomb complex protein BMI-1 | P07948 | Tyrosine-protein kinase Lyn |
| P31946 | 14-3-3 protein beta/alpha | P51668 | Ubiquitin-conjugating enzyme |
| P06241 | Tyrosine-protein kinase Fyn | O60216 | Double-strand-break repair protein rad21 homolog |
| P35222 | Catenin beta-1 | P68104 | Elongation factor 1-alpha 1 |
| Q504Q3 | PAN2-PAN3 deadenylation complex catalytic subunit PAN2 | Q01844 | RNA-binding protein EWS |
| Q9UQL6 | Histone deacetylase 5 | Q96SB4 | SRSF protein kinase 1 |
| Q15022 | Polycomb protein SUZ12 | P19838 | Nuclear factor NF-kappa-B p105 subunit |
| P32121 | Beta-arrestin-2 | P38936 | Cyclin-dependent kinase inhibitor 1 |
| P48431 | Transcription factor SOX-2 | Q14197 | Peptidyl-tRNA hydrolase ICT1, mitochondrial |
| Q92793 | CREB-binding protein | P16104 | Histone H2AX |
| P60709 | Actin, cytoplasmic 1 | Q13501 | Sequestosome-1 |
| P00519 | Tyrosine-protein kinase ABL1 | Q96GG9 | DCN1-like protein 1 |
| P62258 | 14-3-3 protein epsilon | P06493 | Cyclin-dependent kinase 1 |
| P54253 | Ataxin-1 | Q16637 | Survival motor neuron protein |
| P15336 | Cyclic AMP-dependent transcription factor ATF-2 | P09874 | Poly polymerase 1 |

Table S2：The enrichment results of five sub-networks in the HL-expanded network

| Sub-network 1 (68 Nodes) | |
| --- | --- |
| MF | None |
| BP | Developmental maturation  Dendrite development |
| KEGG | None |
| Sub-network 2 (223 Nodes) | |
| MF | Cadherin binding involved in cell-cell adhesion  Protein binding involved in cell-cell adhesion  Protein binding involved in cell adhesion  Cadherin binding |
| BP | Cell cycle phase transition  Mitotic cell cycle phase transition  Negative regulation of epidermal growth factor receptor signaling pathway Negative regulation of ERBB signaling pathway |
| KEGG | Epstein-Barr virus infection  Protein processing in endoplasmic reticulum  Gap junction  Human papillomavirus infection |
| Sub-network 3 (119 Nodes) | |
| MF | Receptor signaling protein activity  Protein tyrosine kinase activity  Receptor signaling protein serine/threonine kinase activity  Protein serine/threonine kinase activity |
| BP | Positive regulation of kinase activity  Positive regulation of protein kinase activity  Immune response-regulating signaling pathway  Regulation of protein serine/threonine kinase activity |
| KEGG | NF-kappa B signaling pathway  Neurotrophin signaling pathway  T cell receptor signaling pathway  MAPK signaling pathway |
| Sub-network 4 (126 Nodes) | |
| MF | Transcription factor binding  RNA polymerase II transcription factor binding  Chromatin binding  Transcriptional activator activity  RNA polymerase II transcription regulatory region sequence-specific binding |
| BP | Histone modification  Peptidyl-lysine modification  Cellular response to organic cyclic compound  Reproductive structure development |
| KEGG | Pathways in cancer  HTLV-I infection  Colorectal cancer  Hepatitis B |
| Sub-network 5 (5 Nodes) | |
| MF | None |
| BP | Interstrand cross-link repair  Regulation of response to DNA damage stimulus  DNA damage response  Detection of DNA damage  Regulation of DNA metabolic process |
| KEGG | None |

Table S3: Uniprot ID of 49 key proteins and their related information in the HL-extended network

| **Uniprot** | **Source** | **Sub-network** | **Name** | **Possible Function in HL** |
| --- | --- | --- | --- | --- |
| Q15717 | Extended^*^ | Sub-network 1 | ELAV-like protein 1 | Developmental maturation of HL cell |
| P54259 | Extended | Sub-network 1 | Atrophin-1 |
| P05067 | Extended | Sub-network 1 | Amyloid-beta precursor protein |
| P0CG48 | Extended | Sub-network 2 | Polyubiquitin-C | Promotion of HL cell motion |
| P63104 | Extended | Sub-network 2 | 14-3-3 protein zeta/delta |
| P02751 | Extended | Sub-network 2 | Fibronectin |
| P07900 | Collected^#^ | Sub-network 3 | Heat shock protein HSP 90-alpha | Participation of signal pathways involved in HL |
| P00519 | Extended | Sub-network 3 | Tyrosine-protein kinase ABL1 |
| Q12933 | Extended | Sub-network 3 | TNF receptor-associated factor 2 |
| P06241 | Extended | Sub-network 3 | Tyrosine-protein kinase Fyn |
| P27986 | Extended | Sub-network 3 | Phosphatidylinositol3-kinase regulatory subunit alpha |
| P19174 | Extended | Sub-network 3 | 1-phosphatidylinositol 4,5-bisphosphate phosphodiesterase gamma-1 |
| P04626 | Extended | Sub-network 3 | Receptor tyrosine-protein kinase erbB-2 |
| P16333 | Extended | Sub-network 3 | Cytoplasmic protein NCK1 |
| Q99683 | Collected | Sub-network 3 | Mitogen-activated protein kinase kinase kinase 5 |
| P34932 | Collected | Sub-network 3 | Heat shock 70 kDa protein 4 |
| P07948 | Extended | Sub-network 3 | Tyrosine-protein kinase Lyn |
| Q9Y6K9 | Extended | Sub-network 3 | NF-kappa-B essential modulator |
| Q13114 | Extended | Sub-network 3 | TNF receptor-associated factor 3 |
| O60674 | Extended | Sub-network 3 | Tyrosine-protein kinase JAK2 |
| P04049 | Extended | Sub-network 3 | RAF proto-oncogene serine/threonine-protein kinase |
| Q05397 | Extended | Sub-network 3 | Focal adhesion kinase 1 |
| P25054 | Collected | Sub-network 4 | Adenomatous polyposis coli protein | Transcription factor binding associated with HL |
| Q96AA8 | Collected | Sub-network 4 | Janus kinase and microtubule-interacting protein 2 |
| P04637 | Extended | Sub-network 4 | Cellular tumor antigen p53 |
| Q14134 | Extended | Sub-network 4 | Tripartite motif-containing protein 29 |
| P35222 | Extended | Sub-network 4 | Catenin beta-1 |
| P35713 | Collected | Sub-network 4 | Transcription factor SOX-18 |
| O14640 | Extended | Sub-network 4 | Segment polarity protein dishevelled homolog DVL-1 |
| O43521 | Extended | Sub-network 4 | Bcl-2-like protein 11 |
| P12931 | Extended | Sub-network 4 | Proto-oncogene tyrosine-protein kinase Src |
| P47928 | Collected | Sub-network 4 | DNA-binding protein inhibitor ID-4 |
| P09086 | Collected | Sub-network 4 | POU domain, class 2, transcription factor 2 |
| Q969V6 | Collected | Sub-network 4 | Myocardin-related transcription factor A |
| O43684 | Extended | Sub-network 4 | Mitotic checkpoint protein BUB3 |
| Q9UGN5 | Collected | Sub-network 4 | Poly polymerase 2 |
| P10071 | Collected | Sub-network 4 | Transcriptional activator GLI3 |
| Q9H165 | Collected | Sub-network 4 | B-cell lymphoma/leukemia 11A |
| Q92794 | Collected | Sub-network 4 | Histone acetyltransferase KAT6A |
| Q92793 | Extended | Sub-network 4 | CREB-binding protein |
| P23769 | Collected | Sub-network 4 | Endothelial transcription factor GATA-2 |
| O15169 | Collected | Sub-network 4 | Axin-1 |
| Q99708 | Collected | Sub-network 4 | DNA endonuclease RBBP8 |
| Q9Y6X2 | Collected | Sub-network 4 | E3 SUMO-protein ligase PIAS3 |
| P21675 | Collected | Sub-network 4 | Transcription initiation factor TFIID subunit 1 |
| Q13287 | Extended | Sub-network 4 | N-myc-interactor |
| O76064 | Extended | Sub-network 5 | E3 ubiquitin-protein ligase RNF8 | Mediation of DNA damage led to HL |
| O96017 | Extended | Sub-network 5 | Serine/threonine-protein kinase Chk2 |
| P0CG47 | Extended | Sub-network 5 | Polyubiquitin-B |

* Extended means that the protein is obtained using expansion strategy.

# Collected means that the protein is obtained by manual collection.

Table S4 Proteins mediated directly and indirectly by five core miRNAs and the possible functions of miRNA in HL.

| miRNA  name | Proteins regulated directly | Proteins regulated indirectly | Possible functions in HL |
| --- | --- | --- | --- |
| miR-335 | O15169、P21675、P47928、O60674、P19174、Q14134、P63104、P35713、Q96AA8 | P12931、P34932、Q13114、P00519、P05067、P04626、Q15717、Q05397、P04637、P35222、P07900、P0CG47、Q92793、P25054、O14640  P27986、Q99683、O43684、P06241、P07948、P04049、P16333、Q9H165、P02751、P63104、Q14134、P19174、O60674、P21675、O15169  P47928 | 1、Epstein-Barr virus infection  2、ErbB signaling pathway  3、Focal adhesion |
| miR-92a | O15169、O43521、P19174 | P02751、P04637、P05067、P35222、P34932、P21675、Q15717、P07900、P00519、P16333、P12931、O76064、P63104、Q92793、P0CG47  Q14134、P25054、P04626、O14640、P54259、P27986、P07948、O60674、P04049、Q13287、P06241、Q05397、Q99683、P35713、P10071  P47928、O43684、P23769、Q9Y6X2、Q92794、O43521、O15169 | 1、VEGF signaling  2、Transcription factor binding associated with H |
| miR-26b | P23769、Q13114、O96017、P02751、P34932、P16333、P0CG47 | P07900、Q15717、P63104、P35222、Q14134、P04637、O15169、Q05397、P19174、O43684、P00519、P12931、P06241、P05067、P07948  O43521、O76064、Q92793、P21675、P04049、P27986、Q99708、P04626、O60674、Q99683、P54259、P35713、P25054、Q92794、P02751  P34932、P0CG47、P23769、O96017 | 1、Viral carcinogenesis  2、Sphingolipid signaling pathway  3、Participation of signal pathways involved in HL  4、Mediation of DNA damage led to HL |
| let-7b | P00519、P07900、P07948、P63104 | P02751、P12931、P34932、Q13114、Q15717、P05067、P16333、O76064、Q92793、P27986、P04626、P04049、O43521、Q99683、P25054  Q13287、P0CG47、P04637、Q14134、P06241、O60674、P54259、O43684、P35222、P19174、Q05397、P21675、P35713、Q9Y6X2、Q969V6  P07900、P00519、P63104、P07948 | 1、ErbB signaling pathway  2、Focal adhesion |
| miR-16 | P05067、P07900、O60674  P27986、P04049、P04637 | P02751、P63104、Q15717、Q92793、P04626、P34932、O43521、P0CG47、P25054、P00519、P35222、O15169、O14640、P12931、Q99683  P06241、P16333、O43684、P07948、Q05397、O96017、P21675、P23769、P54259、P47928、P05067、P04637、P07900、P04049、P27986  O60674 | 1、Developmental maturation of HL cell  2、Viral carcinogenesis, |

Table S5: Number of PPI data extracted from five databases and the database version

| PPI Database | Version of Database | Number of PPI |
| --- | --- | --- |
| BioGrid | v3.4 | 113,073 |
| IntAct | V4.2.6 | 422,82 |
| HPRD | Release 9 | 36,867 |
| MINT | Beta version | 17,524 |
| DIP |  | 3,348 |
| Total |  | 146,295 |

Figure S1:


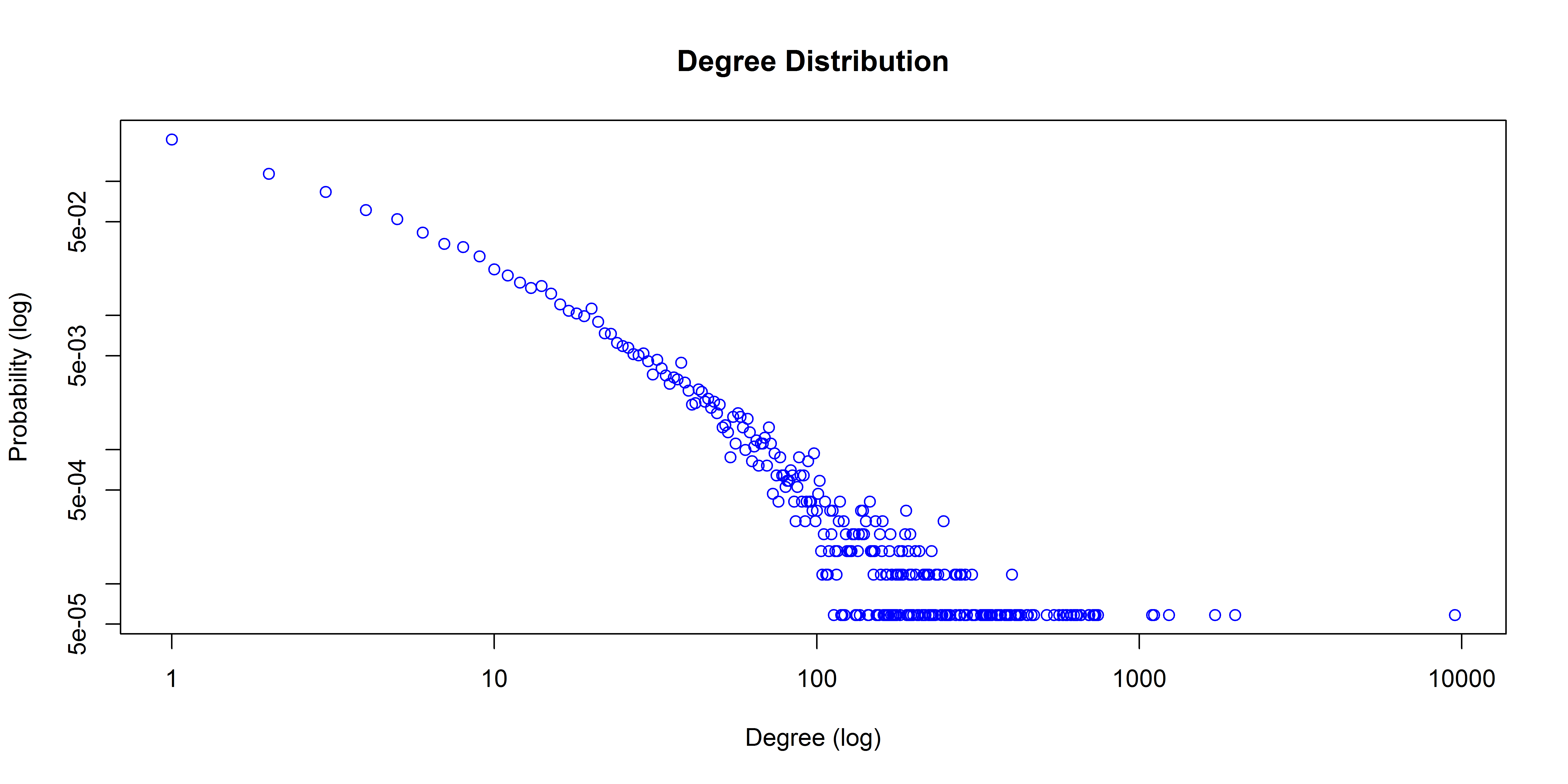


Degree distribution of PPI background network
